# Development and content validation of a Greek electronic tool for the process for nutrition and dietetic practice (e-PNDP): a mixed-methods study in Cyprus

**DOI:** 10.3389/fnut.2026.1831101

**Published:** 2026-06-25

**Authors:** Elina Polydorou, Stella A. Nicolaou, Antonis Zampelas, Eleni P. Andreou

**Affiliations:** Department of Life Sciences, School of Life and Health Sciences, University of Nicosia, Nicosia, Cyprus

**Keywords:** dietetic documentation, digital health, electronic health records, nutrition care process, process for nutrition and dietetic practice

## Abstract

**Background:**

Standardized documentation frameworks such as the Nutrition Care Process (NCP) and the Process for Nutrition and Dietetic Practice (PNDP) aim to improve consistency of clinical reasoning, transparency of decision-making, and patient-centered care in dietetics. However, implementation remains inconsistent and is often limited by time constraints, insufficient applied training, and inadequate integration within digital health systems.

**Methods:**

A sequential three-phase mixed-methods design was employed to develop and validate a Greek electronic PNDP tool (e-PNDP) for routine dietetic practice in Cyprus. In Phase 1, an exploratory national web-based survey was conducted among nutrition and dietetic professionals (*n* = 63) to examine awareness, implementation patterns, and perceived barriers related to structured documentation. In Phase 2, the electronic PNDP tool was developed based on these findings and aligned with the PNDP framework. In Phase 3, the tool underwent expert content validation and usability evaluation by a purposive multidisciplinary panel (*n* = 7). Panel members rated relevance, clarity, and simplicity using a 5-point Likert scale, and item-level and scale-level content validity indices (I-CVI and S-CVI) were calculated.

**Results:**

Survey findings demonstrated high awareness of structured documentation frameworks but variability in sustained implementation, with time burden and challenges in nutrition diagnosis formulation identified as key barriers. Expert evaluation indicated strong content validity across tool modules (I-CVI range: 0.86–1.00; S-CVI/Ave = 0.92) and consistently high usability ratings for relevance, clarity, and simplicity. Qualitative feedback suggested minor refinements related to wording clarity, interface presentation, and inclusion of selected clinical fields, without requiring structural modifications to the PNDP framework.

**Conclusions:**

The electronic PNDP tool (e-PNDP) demonstrated strong content validity and usability, supporting its feasibility as a structured documentation solution within the Cypriot healthcare context. Integration within existing digital workflows and targeted training—particularly in nutrition diagnosis formulation—may enhance sustainable implementation and contribute to improved transparency and standardization of dietetic documentation.

## 1 Introduction

Standardized frameworks are central to delivering consistent, patient-centered nutrition care and to making clinical reasoning visible through clear and systematic documentation. Such frameworks exist within the field of nutrition and dietetics, most notably the Nutrition Care Process (NCP) and its derivative counterpart, the Process for Nutrition and Dietetic Practice (PNDP). These frameworks comprise structured stages—assessment, diagnosis, intervention, and monitoring and evaluation—which support evidence-based practice and accountable clinical decision-making (1, 2). Both are embedded within international professional standards (3–5).

While the NCP and PNDP provide structured models for clinical reasoning and care delivery, documentation represents only one component of these frameworks, serving as the mechanism through which clinical decisions are recorded and communicated. However, implementation remains uneven across settings and countries, with persistent barriers including time constraints, limited applied training, variable professional support, and insufficient integration within digital health systems (6, 7). Across international dietetic contexts, these barriers are frequently associated with manual documentation formats, whereas electronic implementation—particularly integration into electronic health records (EHRs)—has been consistently linked to improved adoption (6, 8, 9). Specifically, the use of predefined templates, guided diagnostic structures such as PES statements, and standardized terminology within EHRs has been associated with improved documentation clarity, workflow efficiency, auditability, and practitioner confidence.

A critical component of structured documentation is the use of standardized language (SL), which underpins consistency, comparability, and clarity in clinical communication. Despite its recognized importance, SL adoption remains variable and is often insufficiently integrated into routine practice.

Beyond structural and technological barriers, implementation of structured documentation frameworks is influenced by a broader range of factors, including practitioner attitudes, behavioral capability, organizational culture, and institutional support. Evidence from implementation science highlights that successful adoption depends not only on tool availability but also on motivation, perceived usefulness, workflow integration, and the presence of supportive training environments. Additionally, interprofessional dynamics and system-level priorities may further influence the extent to which structured documentation is consistently applied in clinical practice (9).

Within the Cypriot healthcare context, these challenges are compounded by the absence of locally developed and validated electronic tools aligned specifically with the PNDP framework. Although electronic record-keeping platforms exist in both public and private sectors, documentation typically relies on unrestricted free-text entry without predefined PNDP structure. This contributes to variability in practice, reduced comparability across practitioners, and limited capacity for systematic monitoring of documentation quality or care outcomes (10). In this context, the developed tool was designed to be compatible with general clinical documentation requirements within the GHS framework, thereby supporting potential integration into existing healthcare systems.

The organization of nutrition and dietetic services in Cyprus spans both the public and private healthcare sectors. Within the public system, services are primarily delivered through the General Health System (GHS), which provides universal coverage and includes dietetic services across primary care, outpatient clinics, and hospital settings. However, a substantial proportion of dietitians also operate in private practice, often independently or within multidisciplinary clinics.

Documentation practices vary across settings, with limited standardization and frequent reliance on unstructured or semi-structured formats. While electronic health record systems are implemented within the GHS, these do not currently incorporate structured PNDP-aligned templates. In private practice, documentation is often maintained using standalone digital tools or paper-based records. To date, no unified legal or regulatory framework mandates a standardized structure for dietetic documentation, further contributing to variability in practice.

These patterns are especially relevant in Cyprus, where structured digital tools for PNDP are not yet validated and documentation capabilities within existing systems may be constrained. Against this background, the present study describes the development and validation of an electronic PNDP format, aiming to produce a usable, standardized documentation solution aligned with professional frameworks and responsive to real-world barriers identified in implementation research.

Despite increasing international and local recognition of the PNDP framework, several critical gaps remain evident in both the literature and clinical practice. First, substantial variability in structured PNDP documentation persists within national contexts such as Cyprus, where implementation patterns have not been systematically examined. Second, although digital integration of structured documentation frameworks is consistently associated with improved documentation quality, sustainability, and workflow efficiency, no locally developed or validated electronic PNDP tools currently exist in the Greek language tailored to the Cypriot healthcare system. Third, documentation format has rarely been conceptualized as an independent implementation determinant influencing clinical reasoning transparency, adoption fidelity, and professional visibility.

To ensure relevance to real-world clinical conditions in Cyprus, the development of the eΔΔΔ*Φ*was preceded by a national baseline needs assessment. This exploratory phase systematically examined awareness, knowledge, and implementation patterns of structured documentation processes among nutrition and dietetic professionals. Findings from this assessment confirm variability in sustained PNDP use, and associated barriers to its implementation such as diagnosis-related challenges, time constraints, and incomplete digital structuring within existing documentation systems. These insights directly informed the structural design, diagnostic scaffolding, and usability priorities of the development of the Greek electronic PNDP tool (eΔΔΔ*Φ*), ensuring that its development was grounded in identified implementation needs rather than theoretical assumptions.

Finally, methodological approaches within the existing literature have predominantly relied on cross-sectional surveys assessing awareness or attitudes, with limited multi-phase development studies integrating empirical needs assessment with structured digital tool design, formal content validation, and usability evaluation.

Addressing these interconnected gaps across practice, digital infrastructure, conceptual alignment and methodology, requires an empirically informed, context sensitive approach. Accordingly, the aim of this study was to develop and formally validate a Greek electronic PNDP tool (e-ΔΔΔ*Φ* or e-PNDP; used interchangeably), grounded in a national baseline needs assessment and evaluated through structured expert consensus, to ensure contextual relevance, structural clarity, and implementation feasibility within the Cypriot healthcare system.

## 2 Methods

This study employed a sequential multi-phase design grounded in a pragmatic research philosophy. Pragmatism was selected as it enables the integration of quantitative and qualitative approaches to address applied research questions related to implementation and usability. The study combined an exploratory needs assessment, structured development of a digital documentation tool (e-ΔΔΔ*Φ*), and formal expert content validation with training to ensure both empirical grounding and methodological rigor.

The research was conducted in three consecutive phases:

• Phase 1: Exploratory national needs assessment of PNDP knowledge and implementation patterns

• Phase 2: Development of a Greek electronic PNDP tool (e-ΔΔΔ*Φ*)

• Phase 3: Expert content validation and usability evaluation through training

**Figure 1:**
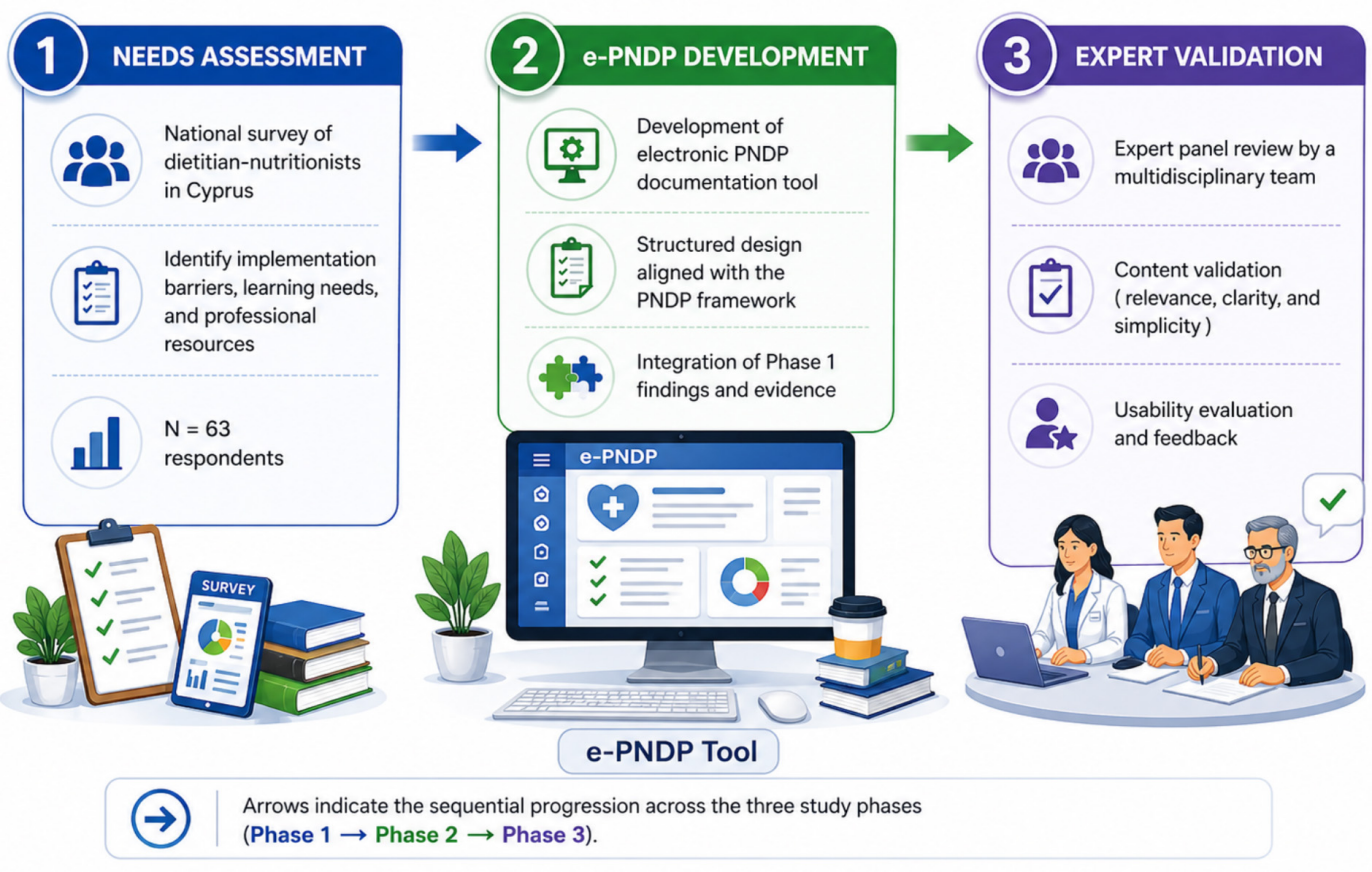
Graphical abstract of the study design and development process of the electronic Process for Nutrition and Dietetic Practice tool (e-PNDP). Overview of the three-phase approach used for the development and validation of the electronic Process for Nutrition and Dietetic Practice (e-PNDP) documentation tool. The study comprised: (1) a national needs assessment survey of dietitian-nutritionists in Cyprus (n = 63) to identify implementation barriers and professional needs; (2) development of the electronic PNDP documentation tool informed by these findings and aligned with the PNDP framework; and (3) expert content validation and usability evaluation by a multidisciplinary panel assessing relevance, clarity, and simplicity. Arrows indicate the sequential progression across the study phases.

This phased design enabled identification of implementation needs, development of a context-adapted digital documentation tool, and its subsequent validation by domain experts. The overall study design and development process of the electronic PNDP tool are illustrated in the graphical abstract and in Figure 1.

### 2.1 Phase 1—Exploratory national needs assessment

#### 2.1.1 Study design, participants and recruitment

Phase 1 employed an exploratory cross-sectional web-based survey to assess national awareness, knowledge, and implementation patterns of the Process for Nutrition and Dietetic Practice (PNDP) among nutrition and dietetic professionals in Cyprus, with particular attention to structural documentation gaps in routine practice. The questionnaire was developed by the research team and informed by established professional frameworks, including the Nutrition Care Process (NCP) model of the Academy of Nutrition and Dietetics, which comprises the four stages of assessment, diagnosis, intervention, and monitoring and evaluation (2, 5). Additional guidance was drawn from the PNDP framework published by the British Dietetic Association (BDA), which operationalises NCP principles within European practice contexts, as well as documentation harmonization initiatives promoted by the European Federation of the Associations of Dietitians (EFAD). Item development was further informed by published implementation research examining facilitators and barriers to the adoption of structured documentation processes in dietetic practice (9).

Although recruitment was conducted at a national level, the achieved sample is not intended to be statistically representative of the entire professional population. The sample predominantly reflects early-career professionals, including postgraduate trainees, and therefore provides insight into emerging practice patterns rather than fully established national trends.

#### 2.1.2 Development of the phase 1 instrument (national needs assessment)

The questionnaire was conceptually grounded in established professional frameworks and adapted linguistically and operationally to the Cypriot healthcare context. As the instrument was developed for exploratory needs assessment, it was not designed as a psychometrically validated scale. The questionnaire was pilot tested with five dietitians to ensure clarity and content relevance prior to distribution.

The questionnaire was developed in Greek to ensure linguistic and contextual appropriateness for the target population. The full instrument is provided as Supplementary material in its original language. To facilitate reproducibility, the questionnaire structure, domains, and response formats are described below.

The instrument consisted of multiple sections designed to capture both quantitative and qualitative data. These included: (i) demographic and professional characteristics; (ii) familiarity with structured documentation frameworks (PNDP, NCP, and SOAP); (iii) self-perceived knowledge of documentation processes; (iv) extent of implementation in routine practice; and (v) perceived barriers, facilitators, and step-specific challenges across the PNDP stages (assessment, diagnosis, intervention, and monitoring and evaluation).

Items were structured using categorical, multiple-choice, Likert-type, and open-ended formats to capture both quantitative implementation patterns and qualitative contextual insights. The estimated completion time was approximately 5–10 min.

Participants were recruited using convenience sampling through dissemination channels including the Cyprus Nutrition and Dietetic Association (CyDNA), professional mailing lists, and targeted posts on professional social media platforms. The survey was administered via Google Forms and distributed through a QR code or direct link. Participation was voluntary and responses were collected anonymously unless participants opted to provide contact details for follow-up phases.

Data collection was conducted between 28 November 2022 and 10 May 2025. Recruitment was implemented in two waves to increase participation within the relatively small professional population of nutrition and dietetic practitioners in Cyprus, while maintaining identical survey instruments and dissemination procedures. A total of 63 complete responses were obtained and included in the analysis.

Survey data were exported from Google Forms and analyzed using IBM SPSS Statistics (version 27). Descriptive statistics were used to summarize frequencies and proportions. Due to the modest sample size and sparse contingency tables, associations between categorical variables were examined using Fisher–Freeman–Halton exact tests with Monte Carlo estimation (10,000 replications; 99% confidence interval). Effect sizes were calculated using Cramer's V, with statistical significance set at *p* < 0.05. Adjustments for multiple comparisons were applied using Bonferroni correction or the Benjamini–Hochberg procedure where appropriate.

Open-ended responses were analyzed using descriptive thematic coding to identify recurring themes related to documentation practices and barriers. When participants endorsed multiple themes, binary coding (1 = selected; 0 = not selected) was used to map frequencies. Composite responses were excluded from individual category counts to avoid inflation of frequencies, and crosstabulations were used descriptively where assumptions for inferential testing were not met.

Findings from Phase 1 were used to identify implementation needs and inform the development of the electronic PNDP tool (e-PNDP) in Phase 2, rather than to generate nationally representative estimates of practice patterns.

### 2.2 Phase 2—Development of the Greek electronic PNDP tool (e-ΔΔΔ*Φ*)

Phase 2 involved the structured development of the e-ΔΔΔ*Φ*, designed to operationalise the Process for Nutrition and Dietetic Practice (PNDP) within the Cypriot healthcare context. The development process followed a user-informed and implementation-oriented approach, integrating empirical findings from Phase 1 with established PNDP framework principles to ensure contextual relevance, conceptual fidelity, and practical usability.

The development of the e-ΔΔΔ*Φ* preserved full alignment with the four-step PNDP framework—assessment, diagnosis, intervention, and monitoring and evaluation—while translating its sequential reasoning into a structured digital documentation format that reflects routine clinical decision-making processes rather than isolated data entry. Particular emphasis was placed on usability-oriented simplicity to facilitate completion within realistic consultation timeframes and reduce documentation burden identified during the Phase 1 needs assessment.

Greek linguistic adaptation of the e-ΔΔΔ*Φ* constructs was undertaken to ensure terminological accuracy and professional appropriateness within the local practice environment, while maintaining conceptual equivalence with the original NCP/PNDP terminology.

Modular data-entry fields were incorporated to capture anthropometric, biochemical, clinical, and dietary domains in a structured and standardized manner. Structured PAS (Problem–Etiology–Signs/Symptoms) options were embedded within the diagnostic component to support systematic formulation of nutrition diagnoses and clinical reasoning. Free-text fields were intentionally limited to reduce documentation variability and cognitive burden while retaining limited narrative flexibility where contextual clinical explanations were required.

The resulting prototype was developed as a standalone digital documentation tool with potential scalability and future integration into the national electronic health record (EHR) infrastructure of the Cyprus General Health System (GHS). Although not directly embedded within the GHS platform, the tool was designed to support interoperability and structured data extraction for future audit, quality monitoring, and outcome evaluation through generation of a standardized PDF documentation output compatible with existing patient record systems.

In addition to alignment with the PNDP framework, the development process was informed by international best practices in structured clinical documentation and digital health tool design, including principles of usability, interoperability, and standardized terminology integration. This ensured that the tool is not only contextually relevant but also consistent with broader international trends in digital dietetic practice.

#### 2.2.1 Technical architecture of the e-ΔΔΔ*Φ* tool

The e-ΔΔΔ*Φ* tool was developed as a structured electronic documentation template designed to operationalise the sequential logic of the Process for Nutrition and Dietetic Practice (PNDP) within routine clinical workflows. The prototype consists of a modular digital form in which data-entry fields correspond to the four PNDP stages: assessment, diagnosis, intervention, and monitoring and evaluation.

Structured input fields and predefined response options were incorporated to guide systematic documentation and reduce reliance on unrestricted free-text entries while retaining limited narrative fields for contextual clinical notes where necessary. Information entered by the practitioner is organized into predefined sections that automatically generate a structured summary of the nutrition care process.

Completed documentation can be exported as a standardized PDF file, enabling storage within patient records and facilitating upload into existing electronic health record systems of the Cyprus General Health System (GHS). The design prioritized usability and compatibility with existing documentation workflows while allowing potential future integration into broader digital health infrastructures.

### 2.3 Phase 3—Expert content validation and usability evaluation

A purposive expert panel was recruited to evaluate the content validity and usability of the e-ΔΔΔ*Φ* tool through an online survey with embedded training components distributed via Google Forms. Purposive sampling was used to ensure that participants possessed advanced professional expertise and familiarity with structured documentation frameworks.

Eligibility criteria required experts to be: (a) registered dietitians or nutritionists with a minimum of 5 years of clinical experience and demonstrated familiarity with the PNDP framework and its diagnostic logic; (b) professionals involved in academic or leadership roles relevant to nutrition and health sciences (e.g., immunologists, statisticians, biochemists); or (c) professionals with advanced roles in the field of nutrition and dietetics. This approach ensured that evaluations reflected both practical clinical experience and broader professional oversight perspectives.

Experts were selected using purposive sampling to ensure inclusion of individuals with advanced knowledge of structured documentation frameworks and their clinical application. To enhance the breadth of perspectives and reduce potential selection bias, the panel included professionals from diverse roles, including clinical practice, academia, and related health sciences. While purposive sampling may introduce selection bias, it is considered appropriate for content validation studies where domain-specific expertise is required.

The validation survey was conducted between January and February 2026. As with Phase 1, because the survey platform (Google Forms) did not record page views, partial completions, or unique visitors, participation and completion rates could not be calculated. Analyses were therefore restricted to fully completed questionnaires. Analyses were restricted to fully completed questionnaires. No IP tracking or duplicate-entry prevention mechanisms were implemented.

Experts independently reviewed the e-ΔΔΔ*Φ* tool and evaluated each module according to relevance, clarity, and simplicity using a structured five-point Likert scale, where higher scores indicated stronger agreement. This multidimensional evaluation ensured that the tool was assessed for conceptual accuracy, structural coherence, and practical usability within routine clinical workflows.

Seven experts completed the validation process. Methodological guidance suggests that panels of five to 10 experts are sufficient for content validity assessment while maintaining feasibility and statistical reliability (11). Quantitative content validity indices (CVI) were calculated at both the item level (I-CVI) and scale level (S-CVI) to quantify expert agreement. Ratings of 4 or 5 on the five-point Likert scale were considered indicative of item relevance and were used for the calculation of I-CVI values. Qualitative feedback was also collected to inform iterative refinements. When six or more experts are included, an I-CVI threshold of ≥0.78 is considered acceptable for demonstrating agreement beyond chance (12, 13). The panel size in the present study therefore meets recommended methodological criteria for content validity estimation.

Scale-level validity was evaluated using two complementary indices. The scale-level content validity index based on the average method (S-CVI/Ave) was calculated as the mean of I-CVI values across items, while the scale-level content validity index based on universal agreement (S-CVI/UA) represented the proportion of items achieving complete agreement among experts. An S-CVI/Ave value of ≥0.90 was interpreted as indicating strong overall content validity.

In addition to CVI indices, descriptive statistics were calculated to further characterize expert evaluations. For each module and evaluation dimension (relevance, clarity, simplicity), means, standard deviations, and frequency distributions were computed to assess rating consistency and identify potential areas of variability.

Qualitative feedback from open-ended responses was analyzed descriptively and organized into thematic categories, including structural refinements, terminology adjustments, interface improvements, and recommendations for additional content. Suggested modifications were reviewed systematically and incorporated where feasible while maintaining conceptual fidelity to the PNDP framework. Where both quantitative ratings and qualitative feedback indicated potential issues, targeted revisions were implemented.

All statistical analyses were conducted using IBM SPSS Statistics (version 27) and Microsoft Excel. The integration of quantitative indices and qualitative feedback strengthened both the methodological robustness and applied relevance of the e-ΔΔΔ*Φ* tool within the Cypriot healthcare context.

### 2.4 Ethical considerations

Ethical approval for this study was obtained from the University of Nicosia Ethics Committee (UREC/2021/15) prior to study commencement. The study was conducted in accordance with the Declaration of Helsinki. Participation was voluntary, and informed consent was obtained electronically through a mandatory consent item at the beginning of each survey. Responses were collected anonymously unless participants voluntarily provided contact details for follow-up communication or acknowledgment of expert contribution. Only data necessary to fulfill the study objectives were collected. All procedures complied with Regulation (EU) 2016/679 (General Data Protection Regulation, GDPR). Data were stored on password-protected institutional cloud systems accessible only to the research team and retained in accordance with institutional data management policies.

## 3 Results

### 3.1 Phase 1—Exploratory national needs assessment

Participants were predominantly early-career professionals, with a median age of 27 years (IQR: 25–30) and a median professional experience of 6 months (IQR: 2–24). The relatively low level of professional experience reflects the inclusion of postgraduate students undertaking supervised clinical placements. Most participants were practicing in Nicosia (*n* = 27) or Limassol (*n* = 16). Independent private practice was the most frequently reported practice setting (n = 38; 60.3%), followed by collaborative private practice (*n* = 10; 15.9%), home visits (*n* = 7; 11.1%), and institutional settings *(n* = 6; 9.5%). Most participants provided services in urban areas (*n* = 50; 79.4%). Two responses from Cypriot professionals practicing outside Cyprus and one respondent not currently practicing were retained to support the limited achieved sample size. Detailed demographic and professional characteristics are presented in Table 1.

**Table 1:** Demographic and professional characteristics of participants (*n* = 63): median (IQR) version.

Characteristic	Value
Age (years), median (IQR)	28.0 (23.5–28.0)
**Age group, *n* (%)**	
22–25 years	25 (39.7)
26–30 years	23 (36.5)
31–45 years	12 (19.0)
46–59 years	2 (3.2)
≥60 years	1 (1.6)
**City/place of practice**, ***n*** **(%)**	
Nicosia	27 (42.9)
Limassol	16 (25.4)
Larnaca	8 (12.7)
Paphos	5 (7.9)
Famagusta	3 (4.8)
Athens	2 (3.2)
UK	1 (1.6)
Not currently practicing	1 (1.6)
**Primary practice setting**, ***n*** **(%)**	
Private office (independent)	38 (60.3)
Shared practice	12 (19.1)
Home visits	7 (11.1)
Hospital/outpatient clinic	6 (9.5)
Professional experience (months), median (IQR)	15.5 (3.75–60.0)
**Professional experience category**, ***n*** **(%)**	
1 month	9 (14.3)
2–3 months	7 (11.1)
4–6 months	10 (15.9)
7–24 months	10 (15.9)
25–48 months	4 (6.3)
>2 years	23 (36.5)

Values are presented as *n* (%) unless otherwise indicated. Median and interquartile range (IQR) values for age and professional experience were estimated from grouped survey categories using category midpoints. Percentages are based on the total sample (*N* = 63). Locations outside Cyprus represent Cypriot dietitians practicing abroad.

#### 3.1.1 Awareness and use of documentation processes

Nearly all participants (98.4%) reported awareness of at least one documentation process (DP). University seminars (68.3%) and clinical placement (58.7%) were the most frequently reported sources of exposure, while CyDNA educational programs were less commonly endorsed (19.0%).

PNDP was the most frequently reported documentation process used in practice (68.3%). SOAP (11.1%) and NCP (6.3%) were less commonly used. A minority (12.7%) reported awareness of documentation processes but no current use.

#### 3.1.2 Knowledge and extent of implementation

Among participants aware of a documentation process (*n* = 62), 74.2% self-rated their knowledge as “very good.” Most respondents (72.6%) reported implementing a documentation process for all patients, while 14.5% reported selective implementation and 12.9% reported non-use.

Regarding stage completion, 73.0% reported implementing all stages of the selected documentation process, 17.5% reported partial implementation, and 9.5% reported non-use. Minor discrepancies across related items were treated as data consistency variation rather than substantive differences.

#### 3.1.3 Documentation process step implementation and perceived difficulty

Most respondents reported not using SOAP (74.6%) or NCP (76.2%). PNDP non-use was lower. Among PNDP users, step completion rates were high, with strongest endorsement for assessment and diagnosis identification (both >95%). Monitoring and evaluation were the least frequently endorsed step (76.6%). Most participants reported no specific difficult stage. When difficulty was reported, it most commonly concerned the diagnosis and intervention stages, particularly within PNDP and NCP.

#### 3.1.4 Awareness of recommendations and international initiatives

Perceptions regarding whether CyDNA had published documentation process recommendations were divided (50.8% “Yes”; 49.2% “No” after collapsing “I don't know” into “No”). No significant association was observed between age group and this perception (*p* = 0.301; Cramer's V = 0.262). Awareness of EFAD's or BDA or American Academy of Nutrition and Dietetics international consultation initiative was low (25.4%), with no significant association with age (*p* = 0.609; Cramer's V = 0.192).

#### 3.1.5 Standardized language preferences and implementation approaches

Half of participants (50.8%) supported the establishment of a standardized method for recording nutrition and dietetic care, while 36.5% indicated partial feasibility across care stages. A small minority questioned usefulness or feasibility. In multiple-response items assessing standardized language (SL) preferences, PNDP was most frequently endorsed (65.1%), followed by NCP (28.6%), SOAP (23.8%), and IDNT (19.0%).

Preferred clinical implementation strategies emphasized ongoing education and integration into electronic health records. In academic settings, inclusion in the formal curriculum was most frequently supported. Estimated implementation timelines ranged from 1.5 to 50 months, with common estimates between 6–12 months.

Participants generally anticipated improvements in professional visibility, auditability, and care quality with SL adoption, although a substantial proportion selected “I don't know.” Alignment with the Cyprus General Health System (GHS) was the most frequently cited consideration when selecting a standardized language system.

#### 3.1.6 Education exposure and improvement needs

Most participants (68.3%) reported awareness of SL instruction during training. During clinical placement, PNDP was most frequently reported (52.4%), though 22.2% reported no SL use during placement.

Recommendations for improving SL adoption focused primarily on increased education, structured examples, and practical training. Time efficiency and maintenance of current systems were also mentioned.

#### 3.1.7 Nutrition diagnosis knowledge and PNDP challenges

Most participants (61.9%) distinguished medical diagnosis from nutrition diagnosis, while 36.5% selected “Sometimes.” When asked to identify a nutrition diagnosis, responses suggested uncertainty, with 44.4% selecting “none of the above” and 41.3% selecting “weight loss.”

When prompted regarding PNDP components, 81.0% correctly identified all core elements. However, 52.4% reported prior use of PNDP but not current use. Most current or past users did not report difficulty (60.3%), although 27.0% reported challenges. Open-ended responses highlighted time burden and difficulty formulating nutrition diagnoses as the most frequently reported barriers.

### 3.2 Phase 2 and 3—Expert content validation results (I-CVI, S-CVI, usability)

#### 3.2.1 Expert validation of the electronic PNDP tool (questionnaire 2)

Seven experts completed the validation questionnaire; demographic data were intentionally not collected. The expert panel comprised clinical dietitians, nutritionists, and academics specifically immunologists, statistician, biochemists, physical education and nutrition experts. Overall feedback was positive, with suggestions focusing on visual readability (e.g., larger screenshots/fonts), minor wording/terminology refinements, and small content additions (e.g., specific clinical fields and date fields).

#### 3.2.2 Item-level content validity (I-CVI)

The I-CVIs demonstrated strong expert agreement across all evaluation domains (Table 2). For relevance, clarity, and simplicity, I-CVI values ranged from 0.86 to 1.00. All items (100%) exceeded the predefined adequacy threshold of I-CVI ≥0.78, indicating consistent agreement among panel members.

**Table 2:** Scale-level content validity of the electronic PNDP tool (e-ΔΔΔ*Φ*).

Domain	S-CVI/Ave	S-CVI/UA	Min I-CVI	Max I-CVI
Relevance	0.92	0.44	0.86	1
Clarity	0.94	0.56	0.86	1
Simplicity	0.90	0.33	0.86	1
Average across the 3 domains	0.92	0.44	0.86	1

I-CVI, item-level content validity index; S-CVI/Ave, scale-level content validity index calculated using the average method; S-CVI/UA, scale-level content validity index calculated using the universal agreement method. An I-CVI threshold of ≥0.78 was considered acceptable for expert panels comprising six or more experts.

At the scale level, the S-CVI/Ave was 0.92, reflecting high overall content validity of the eΔΔΔ*Φ* (Table 3). The S-CVI/UA was 0.44, indicating that 44% of items achieved universal agreement among all experts. Given the stringent nature of universal agreement criteria and the size of the expert panel (n = 7), this level of agreement is considered methodologically acceptable.

**Table 3:** Item-level content validity of the electronic PNDP tool (e-ΔΔΔ*Φ*).

Module	I-CVI/ave
Front page	0.86
Anthropometry assessment	0.90
Biochemistry assessment	0.90
Medical history	0.86
Medication	0.90
Nutrition and dietetics diagnosis	0.95
Summary of assessment	1.00
Print or save	1.00
Print or save (continued)	0.95

I-CVI/Ave = average item-level content validity index across expert ratings.

#### 3.2.3 Descriptive usability ratings

Descriptive analysis indicated high usability across evaluation domains. The mean relevance score was 4.71 (SD = 0.45), the mean clarity score was 4.64 (SD = 0.52), and the mean simplicity score was 4.58 (SD = 0.60). The relatively small standard deviations suggest consistent expert ratings across domains (Table 4).

**Table 4:** Usability ratings of the electronic PNDP tool.

Evaluation dimension	Mean	SD
Relevance	4.71	0.45
Clarity	4.64	0.52
Simplicity	4.58	0.60

Values represent mean scores and standard deviations (SD) based on expert evaluations using a five-point Likert scale, where higher scores indicate greater agreement regarding relevance, clarity, and simplicity of the tool.

#### 3.2.4 Qualitative feedback

Qualitative feedback from the expert panel yielded four primary categories: (1) structural refinements, (2) terminology adjustments, (3) interface improvements, and (4) additional content suggestions.

Structural refinements primarily involved minor reordering and grouping of fields within modules to enhance logical workflow alignment. Terminology adjustments focused on improving syntax and grammatical errors. Interface improvements included refinements to field spacing, dropdown presentation, and section labeling to improve navigability. Additional content suggestions involved minor prompts to enhance documentation completeness. All proposed modifications were reviewed systematically and incorporated where appropriate, without altering the conceptual structure of the PNDP framework.

#### 3.2.5 Integration of quantitative and qualitative findings

Items receiving comparatively lower I-CVI values were reviewed in conjunction with corresponding qualitative feedback. Where quantitative ratings were accompanied by comments indicating ambiguity or workflow misalignment, targeted revisions were implemented. For modules demonstrating high quantitative agreement but minor qualitative suggestions, refinements were incorporated selectively to improve clarity while preserving structural integrity. This integrative approach ensured that the final version of the electronic PNDP tool demonstrated both strong statistical content validity and practical usability within the Cypriot clinical context.

## 4 Discussion

This study examined national awareness and implementation patterns of documentation processes (DPs) and standardized language (SL) among nutrition and dietetic professionals (NDPs) in Cyprus and subsequently evaluated the content validity and usability of a newly developed e-ΔΔΔ*Φ*. The findings highlight variability in sustained implementation, diagnosis-related challenges, and structural documentation gaps, while expert validation demonstrated strong content validity and high usability of the proposed digital tool.

Although awareness of documentation processes was nearly universal among respondents, implementation patterns were less consistent. PNDP was the most frequently reported framework in practice; however, a substantial proportion of participants indicated discontinuation of use despite prior exposure. This divergence between awareness and sustained implementation suggests that familiarity alone does not ensure routine adoption. Instead, contextual and operational factors appear to influence long-term integration into practice.

Qualitative findings from Phase 1 identified time burden and limited applied training, particularly in diagnostic formulation, as key barriers. These barriers align with the observed difficulties in diagnosis and intervention steps, which were most commonly reported as challenging. Furthermore, responses to the nutrition diagnosis knowledge item revealed uncertainty in identifying appropriate diagnostic statements, despite general conceptual recognition that medical and nutrition diagnoses differ. Collectively, these findings suggest that while the structural logic of PNDP is recognized, applied diagnostic reasoning may remain an area requiring reinforcement.

### 4.1 Interpretation of documentation process adoption in Cyprus

The strong association between DP awareness and university-based exposure underscores the central role of formal education in shaping professional familiarity with structured documentation. PNDP emerged as the dominant framework across both training and reported practice contexts, indicating educational alignment with professional expectations.

However, the transition from academic exposure to sustained clinical implementation appears vulnerable to real-world pressures. The proportion of respondents reporting previous but not current PNDP use suggests that documentation frameworks may be deprioritised when confronted with workload constraints, time limitations, and system-level misalignment. This interpretation is reinforced by participants' emphasis on the need for continuous professional education and integration into electronic records, indicating that structured documentation may be more sustainable when embedded within digital workflows rather than reliant on manual formats.

### 4.2 Observed difficulties in implementation

Diagnosis and intervention steps were consistently identified as the most challenging components of structured documentation. These steps require translation of assessment data into precise, problem-oriented statements and targeted intervention plans, representing higher-order clinical reasoning tasks. The comparatively lower endorsement of monitoring and evaluation steps may reflect documentation fatigue or time pressure, rather than conceptual misunderstanding.

The high proportion of respondents selecting “none of the above” in the forced-choice nutrition diagnosis item further indicates uncertainty in applied diagnostic reasoning. This pattern suggests partial conceptual knowledge but reduced confidence in operationalising PAS statements. These findings support the rationale for embedding structured diagnostic scaffolding within electronic tools, as digital prompts may help reduce cognitive load and standardize reasoning pathways.

### 4.3 Implementation preferences and system-level alignment

Participants overwhelmingly supported introducing SL through continuous professional development and curricular integration, reinforcing the importance of structured educational reinforcement. Notably, when asked to prioritize frameworks for adoption, alignment with the Cyprus General Health System (GHS) was the dominant consideration. This finding highlights that successful nationwide standardization may depend less on conceptual endorsement and more on system-level compatibility and digital infrastructure support.

These results underscore the importance of viewing documentation format not merely as a technical feature but as an implementation determinant. Embedding structured documentation within electronic systems may enhance feasibility, sustainability, and auditability, thereby bridging the gap between theoretical endorsement and routine use.

### 4.4 Expert validation of the electronic PNDP tool

Expert validation demonstrated strong content validity across all modules, with mean I-CVI values ranging from 0.86 to 1.00 and an overall S-CVI/Ave of 0.92. Usability ratings were consistently high across relevance, clarity, and simplicity dimensions, indicating broad professional agreement regarding structural adequacy and practical feasibility.

Qualitative feedback primarily concerned minor structural refinements, terminology adjustments, and interface improvements rather than conceptual redesign. This pattern suggests that the electronic tool was perceived as structurally sound and aligned with PNDP logic, while benefiting from iterative usability optimisation.

Importantly, no module fell below the predefined content validity threshold, supporting the methodological robustness of the development process. The integration of quantitative and qualitative feedback ensured that revisions were both statistically informed and contextually responsive.

### 4.5 Conceptual contribution: documentation format as an implementation determinant

Beyond reporting awareness and usability outcomes, this study makes a conceptual contribution by positioning documentation format—manual vs. structured electronic—as an independent implementation determinant in dietetic practice. Existing literature has largely examined awareness, attitudes, or barriers related to the PNDP/NCP framework without explicitly conceptualizing how the format of documentation itself may influence implementation fidelity, reasoning transparency, and sustainability (11, 12).

Findings from Phase 1 demonstrated that awareness alone does not guarantee sustained use. A substantial proportion of participants reported prior PNDP exposure but current non-use, suggesting that contextual and structural barriers may undermine routine application. Time burden and diagnostic uncertainty were recurrent themes, particularly in relation to the formulation of nutrition diagnoses. These findings support the proposition that structured digital scaffolding may reduce cognitive load and enhance adherence to framework logic (9, 13).

The electronic PNDP tool developed in this study operationalises documentation format as a structured reasoning pathway rather than a passive recording mechanism. By embedding PES scaffolding, modular data fields, and sequential workflow alignment, the tool transforms the documentation interface into an active implementation support mechanism. In this sense, format becomes not merely a technical feature but a structural facilitator of clinical reasoning consistency (14–16).

This reframing contributes to implementation science in dietetics by highlighting the interaction between professional competence, organisational context, and digital infrastructure. It suggests that adoption is not solely dependent on knowledge or attitudes but may be strengthened when structured reasoning is embedded into workflow-compatible electronic systems.

### 4.6 Policy and practice implications for Cyprus

The findings have direct implications for professional regulation, educational programming, and digital health policy within Cyprus.

First, the near-universal awareness of documentation processes indicates that foundational exposure exists within academic curricula. However, the variability in sustained implementation and reported diagnostic difficulties suggests that ongoing professional development initiatives may be required to reinforce applied diagnostic reasoning and structured documentation skills beyond graduation.

Second, participants emphasised alignment with the General Health System (GHS) as a primary determinant for adopting standardised language frameworks. This underscores the importance of system-level integration. Embedding structured PNDP templates within national electronic health platforms may enhance feasibility, promote uniform documentation practices, and facilitate quality-of-care monitoring (8). An additional consideration for implementation is interoperability with existing health information systems. Minimising duplicate data entry through integration with electronic health records is essential to reduce documentation burden and potential errors. Future development should prioritise automated data transfer (e.g., anthropometric, medical, and pharmacological data) from existing clinical systems into the PNDP tool to enhance efficiency and user acceptance.

Third, the validated electronic PNDP tool provides an initial prototype that could serve as a foundation for broader digital transformation initiatives in dietetic care. If integrated within national systems, such a tool could support auditability, interprofessional communication, and potential linkage between documentation quality and reimbursement or performance indicators.

Collectively, these findings support the argument that structured digital documentation should be viewed as a policy-level priority rather than solely an individual practitioner responsibility.

### 4.7 Strengths and limitations

This study employed a sequential multi-phase design integrating a national needs assessment, structured tool development, and formal expert content validation with embedded training components. The integration of quantitative and qualitative data enhanced methodological robustness and enabled triangulation between practitioner experiences and expert consensus. The use of established content validity indices (I-CVI and S-CVI) further strengthens the credibility of the validation process.

Importantly, this study represents the first structured attempt to develop and validate a Greek-language electronic PNDP tool tailored to the Cypriot healthcare context. By grounding tool development in empirical national data, the study ensured contextual responsiveness rather than theoretical transplantation.

Several limitations should be considered when interpreting the findings. The exploratory national needs assessment achieved a modest sample size and was based on convenience sampling, thereby limiting generalisability and introducing potential selection bias. In addition, Phase 1 relied on self-reported practices rather than objective documentation audits, which may have led to overestimation of implementation. The sample was predominantly composed of early-career professionals, which may have influenced reported implementation patterns due to greater exposure to recent academic training but more limited clinical experience. Accordingly, findings should be interpreted as indicative of trends within a specific segment of the workforce rather than representative of the national professional population.

Furthermore, representation of professionals working within the public healthcare system was limited, which may further restrict the applicability of findings across all practice settings.

The expert validation panel, while methodologically adequate in size according to established recommendations, comprised seven purposively selected experts and may not fully capture the diversity of professional perspectives. Although purposive sampling is appropriate for content validation studies, it may introduce selection bias, particularly if participants share similar professional backgrounds or viewpoints. In addition, usability evaluation was limited to expert-based assessment rather than real-world clinical implementation. Future research should therefore evaluate the tool in routine practice settings to assess long-term feasibility, efficiency, and impact on documentation quality.

### 4.8 Future perspectives

Future research should extend beyond content validation to examine real-world implementation outcomes. Pilot integration of the e-ΔΔΔ*Φ* within selected clinical settings would allow assessment of documentation completeness, time efficiency, user satisfaction, and patient-related outcomes.

Longitudinal evaluation studies could investigate whether structured electronic scaffolding improves diagnostic accuracy and monitoring consistency over time. Additionally, comparative research across other small health systems could explore scalability and adaptability of the model.

At a systems level, integration with the national electronic health record platform would enable structured data extraction for quality monitoring and outcome benchmarking. This may create opportunities to link documentation quality with performance metrics, further incentivising structured practice.

Ultimately, advancing digital PNDP integration requires collaboration between professional bodies, academic institutions, and health system policymakers to ensure that structured documentation becomes embedded within routine clinical infrastructure rather than remaining an optional practice enhancement.

## 5 Conclusion

This study provides empirical and methodological support for the development and validation of a Greek electronic PNDP tool (e-ΔΔΔ*Φ*) tailored to the Cypriot healthcare context. Findings from the national needs assessment highlighted variability in structured documentation practices, diagnostic challenges, and implementation discontinuity despite high awareness of documentation processes. These gaps underscored the need for a structured, workflow-aligned digital solution.

Expert validation demonstrated strong content validity and high usability across evaluation domains. Ratings for relevance, clarity, and simplicity consistently exceeded established adequacy thresholds, indicating that the electronic tool is conceptually aligned with the PNDP framework and operationally feasible for routine clinical use. Suggested refinements were minor and primarily focused on usability enhancements, including visual formatting improvements, minor terminology adjustments, and inclusion of select clinically relevant fields (e.g., current weight and date documentation). No structural redesign was required.

Collectively, the findings suggest that a structured electronic PNDP tool can contribute to greater standardisation of dietetic documentation, improved transparency of clinical reasoning, and enhanced consistency in care delivery. Importantly, the sustainability of implementation is likely to depend on integration within existing digital infrastructures, alignment with the General Health System (GHS), and provision of targeted training—particularly in nutrition diagnosis formulation.

## Data Availability

The raw data supporting the conclusions of this article will be made available by the authors, without undue reservation.
